# Research on Electro-Acoustic Synergistic Partial Discharge Detection Technology for Cable Terminations

**DOI:** 10.3390/s26113460

**Published:** 2026-05-30

**Authors:** Cong Chen, Xiaojian Wang, Yanju Li, Qichao Chen

**Affiliations:** 1 China Power Huachuang (Suzhou) Electricity Technology Research Co., Ltd., Suzhou 215123, China; crcn2007@cpibj.com.cn (C.C.); xjwang@cpibj.com.cn (X.W.); 2 State Key Laboratory of High-Efficiency Special Cable Technology, Harbin University of Science and Technology, Harbin 150080, China; 2420310138@stu.hrbust.edu.cn

**Keywords:** cable termination, partial discharge, high-frequency current transformer, Sagnac optical fiber interferometer

## Abstract

To address the limited spatial localization accuracy of partial discharge (PD) in high-voltage cable terminations and the difficulty in accurately determining the trigger time in traditional ultrasonic detection, this paper proposes an electro-acoustic synergistic localization technology based on a high-frequency current transformer (HFCT) and a Sagnac optical fiber interferometer. A high-sensitivity Sagnac acoustic sensor based on a 3D-printed photosensitive resin mandrel was developed. Through structural design and 0–50 kHz amplitude–frequency testing, the sensor exhibits a dominant resonant response at 33.2 kHz. This narrow-band, high-sensitivity characteristic effectively enhances the perception capability for weak PD ultrasonic signals. An electro-acoustic synergistic detection system was constructed, in which the high-frequency PD current signal captured by the HFCT was used as the electrical time reference, and a dual-channel Sagnac sensor array was used to extract the arrival times of ultrasonic waves. In a 12 kV laboratory cable-termination PD experiment, the proposed system identified the representative built-in air-gap PD source with an absolute localization error of 5 mm under the tested laboratory configuration. This value should be interpreted as the localization result for the tested representative defect, rather than as a generally validated accuracy specification of the system. This study provides a proof-of-concept laboratory demonstration of an electro-acoustic localization strategy that combines the fast electrical response of HFCT detection with the electromagnetic-interference immunity and acoustic sensitivity of Sagnac fiber-optic sensing.

## 1. Introduction

With the expansion of urban power grids and the continuous growth in demand for high-capacity power transmission, high-voltage (HV) and extra-high-voltage (EHV) cable systems have become the core infrastructure of modern power networks. Since cable terminations operate long-term in harsh environments characterized by the multi-field coupling of strong electric fields, non-uniform thermal fields, and mechanical stress, their insulation condition is directly related to the operational safety of the power grid. Partial discharge (PD), as the most representative physical indicator in the early stages of insulation degradation, requires high-sensitivity detection and precise spatial localization, which serves as the scientific prerequisite for achieving early warning of cable faults and precise operation and maintenance [1,2,3].

In existing engineering applications, electrical detection methods based on high-frequency current transformers (HFCTs) are widely used for the online PD monitoring of cables and their accessories due to their flexible installation, electrical isolation from primary equipment, and sensitive response to discharge pulse currents [4,5]. Li et al. designed a miniature, high-sensitivity HFCT for PD detection in cable joints, achieving a gain of over 44 dB (150 mV/mA) in the 1 MHz to 5 MHz frequency range, and analyzed the factors affecting sensor sensitivity [6]. However, HFCTs operate by being clamped onto the cable grounding wire to induce pulse currents. Their detection sensitivity heavily relies on the electrical distance between the sensor installation location and local discharge sources (such as cable joints and terminations), as well as the impedance characteristics of the coupling circuit [7]. This dependence leads to severe signal amplitude attenuation and makes the system susceptible to electromagnetic noise generated by other electrical equipment along the propagation path [8]. More importantly, a single HFCT sensor can only provide information on the presence and relative intensity of a discharge; it cannot deduce the physical location of the discharge from a single measurement point, which is the fundamental reason why it cannot achieve direct localization in principle [9]. Therefore, although a single electrical method has the advantage of capturing the starting time of a PD event, it struggles to balance anti-interference capability with the demand for axial localization under complex operating conditions [10].

In recent years, fiber-optic acoustic sensing has shown promising application prospects in the field of PD detection due to its excellent insulation performance, strong electromagnetic interference (EMI) immunity, suitability for strong electromagnetic environments, and capabilities for long-distance transmission and distributed deployment [11,12,13,14,15]. The Sagnac optical fiber interferometer exhibits unique advantages in PD detection, particularly in enhancing sensitivity and EMI resistance. Compared with traditional HFCTs, the Sagnac interferometer detects the ultrasonic signals generated by PD, circumventing the HFCT’s susceptibility to power-frequency current saturation and electromagnetic interference, thereby improving detection accuracy and sensitivity. Li et al. proposed a method applying Sagnac fiber-optic acoustic sensing and Φ-OTDR fiber sensing for PD acoustic detection in cable joints, comparing it with traditional PZT sensors. They verified that the Sagnac system offers a superior signal-to-noise ratio (SNR) and that Φ-OTDR possesses distributed localization capabilities; however, their research focused more on comparing the detection performance of different sensing schemes and verifying localization feasibility, lacking in-depth investigation into acquiring the initial PD time and the high-precision synergistic localization mechanism [16]. Song et al. proposed a PD ultrasonic detection method based on Sagnac interference and significantly improved PD ultrasonic detection sensitivity by establishing a multi-parameter sensitivity model to optimize the optical probe’s material, skeleton height, radius, and fiber winding length. Nevertheless, their study primarily addressed sensor probe performance optimization, without systematically researching the PD source direction discrimination and localization methods themselves [17]. Yu et al. proposed a Sagnac PD ultrasonic detection system with high noise suppression capability, introducing a Faraday mirror and a symmetrical optical path structure to suppress low-frequency vibration noise and polarization noise, thereby improving detection stability in complex environments. However, this research still primarily focused on noise suppression and signal detection enhancement, without further resolving the lack of a starting time reference in PD localization and the directional ambiguity of a single sensing channel [18]. Although pure acoustic detection schemes have good EMI immunity, the lack of an electrical starting time reference strictly synchronized with the PD event often restricts them to using relative arrival information, resulting in insufficient model constraints. When a cable termination is approximated as a one-dimensional linear propagation channel, a single acoustic sensing unit can typically only provide propagation delay information relative to the sensing location, making it difficult to further determine whether the PD source is located to the left or right of the sensing unit, thus creating directional ambiguity.

To further clarify the difference between the proposed method and existing techniques, a concise numerical comparison is provided here. In HFCT-based electrical detection, Li et al. reported a gain of over 44 dB, corresponding to 150 mV/mA in the 1–5 MHz frequency range [6], which indicates the strong capability of HFCTs for capturing high-frequency PD current pulses. However, single-point HFCT detection mainly provides discharge occurrence and relative intensity information, and it is difficult to directly determine the physical position of the PD source. Fiber-optic acoustic methods such as Sagnac and Φ-OTDR sensing have shown good electromagnetic-interference immunity and acoustic detection capability [16,17,18], but acoustic-only methods still lack an absolute electrical starting-time reference for time-of-flight localization. In contrast, the present work focuses on integrating the HFCT electrical time reference with dual-Sagnac acoustic arrival-time detection. Under the tested laboratory cable-termination configuration, the developed Sagnac sensor exhibited a dominant resonant response at 33.2 kHz, and the proposed system identified the representative PD source with an absolute localization error of 5 mm.

The main contribution of this work is the system-level integration of an HFCT-based electrical time reference with a dual-Sagnac resonant acoustic sensing configuration for axial PD localization in cable terminations. The HFCT, Sagnac sensing unit, and time-of-flight calculation are not treated as individually new techniques; rather, their coordinated use is employed to improve the reliability of PD starting-time determination and reduce directional ambiguity in acoustic localization. The novelty of this work lies in the system-level combination of HFCT-based electrical triggering and dual-Sagnac acoustic arrival-time detection for axial PD localization in cable terminations, rather than in the individual HFCT sensor, Sagnac interferometer, or time-of-flight calculation method themselves. Through theoretical analysis and material selection, a 33 kHz resonant mandrel fiber probe based on a photosensitive resin mandrel structure was designed, and a 3 × 3 coupler demodulation algorithm was introduced to ensure high-fidelity signal extraction. Building upon this, a joint localization mathematical model was constructed, utilizing the HFCT as the time zero point and the dual Sagnac sensors to provide acoustic time differences. An experimental platform for partial discharge in cable terminations was built. Through the acquisition of discharge data and electro-acoustic time-difference calculation, the feasibility of the proposed axial localization method was verified under the laboratory cable-termination configuration. This method uses the HFCT signal to provide an electrical reference time and the dual-Sagnac sensing array to reduce the directional ambiguity of acoustic localization, thereby combining the fast response of electrical detection with the EMI immunity of fiber-optic acoustic sensing.

The remainder of this paper is organized as follows. Section 2 introduces the operating principles of the HFCT and Sagnac optical fiber interferometer, and establishes the electro-acoustic joint localization model. Section 3 presents the amplitude–frequency characterization of the mandrel-type Sagnac sensor and the cable-termination PD localization experiment. Section 4 summarizes the main conclusions, discusses the limitations of the present laboratory validation, and outlines future work.

## 2. Sensor Detection Principle

### 2.1. Operating Principle of HFCT

**Figure 1:**
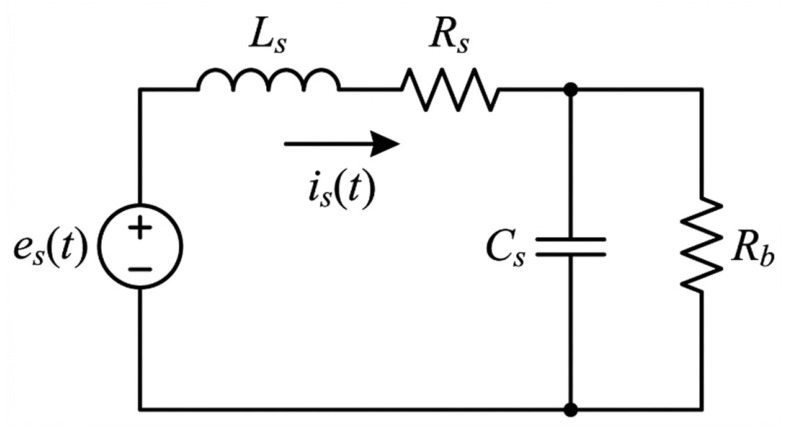
Equivalent circuit model of the HFCT.

A high-frequency current transformer (HFCT) is a passive sensing unit designed based on the principles of electromagnetic induction and transmission line theory, specifically intended to capture megahertz-level high-frequency pulse currents excited during the partial discharge process in power equipment [19]. When a partial discharge occurs within the cable insulation, it excites a steep-front pulse current i_PD_(t), which rapidly propagates along the cable grounding circuit. By non-invasively clamping the HFCT onto the cable grounding wire, the grounding wire carrying the PD pulse current serves as the primary winding of the HFCT; the high-frequency alternating magnetic field generated by the grounding wire passes through the high-permeability magnetic core of the HFCT, thereby inducing a voltage signal corresponding to the PD characteristics in the secondary winding wound on the core, achieving contactless detection of the PD pulse current [20]. To accurately quantify the dynamic response process of the HFCT to PD pulses, its equivalent circuit model is established, as shown in Figure 1.

In this equivalent circuit, the induced electromotive force e_s_(t) on the secondary side of the HFCT can be expressed by the principle of mutual inductance as follows:

(1) $$e_{s}(t)=M\cdot \frac{di_{PD}(t)}{dt}$$

where M is the mutual inductance coefficient between the primary and secondary windings of the HFCT, which is jointly determined by the core permeability, the number of winding turns, and the geometric structure. Considering the high-frequency operating environment of PD pulses, the output response characteristics of the HFCT are not solely determined by the mutual inductance coefficient, but are also comprehensively constrained by the inherent electrical parameters of the secondary winding. Based on Kirchhoff’s Voltage Law, a modified secondary circuit equation for the HFCT can be established to fully describe its high-frequency dynamic response characteristics:

(2) $$L_{s}\frac{di_{s}(t)}{dt}+(R_{s}+R_{b})i_{s}(t)+\frac{1}{C_{s}}\int i_{s}(t)dt=e_{s}(t)$$

where L_s_ is the inductance of the secondary winding; R_s_ is the internal resistance of the winding; C_s_ is the distributed capacitance formed between winding turns and to ground; R_b_ is the matching load resistance on the secondary side; and i_s_(t) is the induced current in the secondary circuit.

From the perspective of circuit characteristics, the distributed capacitance C_s_ and inductance L_s_ of the secondary winding together constitute the second-order resonant system of the HFCT, determining its core band-pass filtering amplitude–frequency characteristics. In frequency-domain analysis, the transfer impedance Z_t_(ω) of the HFCT exhibits typical band-pass characteristics: its lower cut-off frequency f_L_ in the low-frequency band is determined by the formula f_L_ ≈ R_b_/2πL_s_, while the upper cut-off frequency in the high-frequency band is primarily limited by the winding’s distributed capacitance C_s_.

By selecting high-permeability, high-frequency, low-loss magnetic core materials and optimizing the number of winding turns and the winding process to control the distributed capacitance C_s_, the HFCT can achieve a flat and stable transfer impedance over a wide frequency band ranging from several MHz to tens of MHz. This ensures the high-fidelity restoration of the nanosecond-level steep rising edge of the PD pulse signal, avoiding signal waveform distortion.

For the electro-acoustic synergistic PD localization system proposed in this study, the core value of the HFCT lies in its nanosecond-level rapid response capability. Since the propagation speed v_e_ of electromagnetic waves in the cable conductor is close to the speed of light, which is far higher than the propagation speed of ultrasonic waves in the cable’s XLPE insulation medium, the rising edge time t_0_ of the PD pulse detected by the HFCT, after calibration for the system’s fixed hardware delay, can serve as the absolute time reference benchmark for the occurrence of the PD event. This provides a unified time zero point for the subsequent precise extraction of acoustic signal arrival times and time-difference localization calculations.

### 2.2. Principle and Demodulation System of the Sagnac Optical Fiber Interferometer

The Sagnac optical fiber interferometric sensor splits the light emitted by the light source into two beams of equal intensity via a coupler, which then propagate clockwise and counterclockwise along the optical fiber loop, respectively. An interference output is generated when the two beams recombine. If there is no external disturbance, the two counter-propagating light beams experience essentially the same optical path length, and their phase difference remains stable; however, when the ultrasonic waves generated by a partial discharge act upon the sensing fiber, minute changes occur in the fiber’s length and refractive index, thereby causing phase modulation, which ultimately manifests as a change in the interference output [21,22].

**Figure 2:**
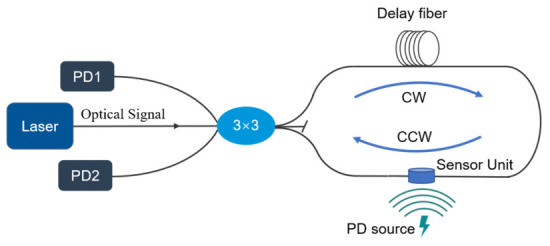
Principle of the Sagnac optical fiber interferometer.

In Figure 2, PD1 and PD2 denote two identical photodetectors used for photoelectric conversion of the optical interference signals. Their output voltage signals are subsequently subtracted in the oscilloscope/data-processing stage to suppress common-mode noise, light-intensity fluctuation, and DC drift.

Let the phase of the light in the sensing fiber be the following:

(3) $$\phi (t)=\frac{2\pi nL}{\lambda }$$

where n is the refractive index of the optical fiber, L is the length of the sensing fiber, and λ is the wavelength of the incident light. In this paper, a symmetrical 3 × 3 optical fiber coupler is introduced into the Sagnac sensing system. The physical structure of the 3 × 3 coupler naturally divides the interference light into three paths, with a fixed 120° phase difference between any two outputs. After conversion by photodetectors, the three output voltage signals can be expressed as follows:

(4) $$V_{k}(t)=D+I_{0}\cos\left[\Delta \phi_{s}(t)+(k-1)\frac{2\pi }{3}\right],\;\;\;k=1,2,3$$

where D is the DC bias component, and I_0_ is the AC amplitude related to the interference fringe contrast. After acquiring these three unbalanced signals with constant phase differences, the system employs a symmetrical passive homodyne demodulation algorithm. First, the DC component D is eliminated. Then, the AC components undergo differentiation and cross-multiplication, followed by integration processing, which can convert the signal containing a cosine nonlinear term into a strictly linear output:

(5) $$V_{out}(t)=K\cdot \Delta \phi_{s}(t)$$

where K is the hardware demodulation gain constant of the system. The demodulation scheme based on the 3 × 3 coupler helps reduce signal fading caused by static phase drift and improves the stability of ultrasonic waveform acquisition, thereby providing more reliable time-domain information for subsequent arrival-time estimation.

For the localization problem in this paper, the key role of the Sagnac sensor lies in acquiring the arrival time of the PD ultrasonic wave. The time at which the k-th Sagnac sensor receives the ultrasonic signal can be expressed as follows:

(6) $$t_{S,k}=t_{0}+\frac{d_{k}}{v}+\tau_{S,k}$$

where t_s,k_ is the acoustic arrival time recorded by the k-th Sagnac sensor, t_0_ is the electrical reference time provided by the HFCT, d_k_ is the acoustic propagation distance from the PD source to the k-th Sagnac sensor, v is the effective axial acoustic velocity in the tested cable-termination structure, and τ_S,k_ is the fixed delay introduced by the corresponding Sagnac sensing and acquisition channel. In this study, v was taken as 1900 m/s. Therefore, the Sagnac sensor essentially provides information on the acoustic propagation of the partial discharge.

It should be pointed out that although a single Sagnac sensor can perceive the arrival of an ultrasonic wave and provide the propagation delay, when approximating the cable as a one-dimensional linear model, it is difficult to determine whether the PD source is located to the left or right of the sensor using only a single unit; thus, there is a directional ambiguity problem. Therefore, the Sagnac sensor is more suitable as an acoustic arrival time detection unit. The determination of the PD starting time and the unique calculation of the spatial position still need to be jointly accomplished by combining the HFCT reference time and the unified information from the dual Sagnac sensors.

### 2.3. Electro-Acoustic Joint Localization Principle

When a partial discharge occurs, the discharge pulse simultaneously excites an electrical signal and an ultrasonic signal. The electrical signal propagates rapidly along the conductor and the grounding circuit and is detected by the HFCT; the ultrasonic signal propagates along the cable structure and is received by two sets of Sagnac sensors, respectively. Because the propagation speed of the electrical signal is much higher than that of the ultrasonic signal, after completing the system’s fixed delay calibration, the detection time of the HFCT can be used as the reference starting time for the PD event. Since the axial length of the cable termination is much greater than its radial diameter, and the ultrasonic wave signals generated by the PD primarily propagate along the cable insulation layer and axial structure, simplifying the entire termination system into a one-dimensional linear system can not only satisfy the requirements for engineering localization accuracy but also significantly reduce the complexity of the localization algorithm.

**Figure 3:**
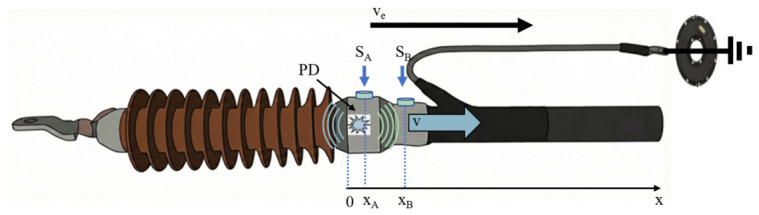
Methodology diagram of the HFCT-assisted dual-Sagnac electro-acoustic joint localization method.

The one-dimensional model used in this work is intended for axial localization of the PD source along the cable termination, rather than for reconstructing the complete three-dimensional acoustic propagation field. In practical cable terminations, multilayer structures may introduce dispersion, reflection, mode conversion, and coupling loss. These effects mainly influence the acoustic amplitude and the later oscillatory components of the received waveform. In this study, the localization calculation is based on the first distinguishable acoustic arrival time under a fixed laboratory configuration, while reflected and delayed components are not used for time-of-flight estimation. Therefore, the one-dimensional approximation is considered suitable for verifying the feasibility of axial electro-acoustic localization. It should be emphasized that the present one-dimensional model is used to verify the axial localization feasibility under a fixed laboratory configuration. Although the dual-Sagnac arrangement can reduce the directional ambiguity of single-sensor acoustic localization, the discrimination rule still needs broader experimental validation for different PD source positions, sensor spacings, and cable-termination structures, see Figure 3.

The localization procedure consists of three steps. First, the rising edge of the HFCT signal is extracted as the electrical reference time of the PD event after fixed-delay compensation. Second, the arrival times of the acoustic signals received by Sagnac-A and Sagnac-B are determined from the first distinguishable rising edges of the acoustic waveforms. Third, the acoustic time-of-flight values are converted into propagation distances using the calibrated acoustic velocity, and the two Sagnac sensors are jointly used to determine the unique axial position of the PD source. In this way, the HFCT provides the absolute time reference, while the dual-Sagnac configuration helps reduce the directional ambiguity of single-sensor acoustic localization under the tested configuration.

Let the starting end of the cable termination stress cone be the coordinate origin x = 0, the actual location of the discharge point be x_pd_, and the rising edge time of the current pulse signal captured by the HFCT sensor be the starting time of the discharge occurrence, t_0_. The times when the two sets of Sagnac sensors detect the ultrasonic signal are t_sA_ and t_sB_, respectively. Assume the system has completed the calibration and compensation of the hardware delay τ_S,k_ for each channel. According to the electro-acoustic synergistic localization principle, the calculated distance d_A_ from the discharge point to Sensor A is as follows:

(7) $$d_{A}=v\cdot (t_{sA}-t_{0})$$

where d_A_ is the acoustic propagation distance from the PD source to Sagnac-A, t_sA_ is the acoustic arrival time of Sagnac-A, t_0_ is the HFCT electrical reference time, and v is the effective axial acoustic velocity. Through redundancy verification with the dual sensors, pseudo-solutions caused by the symmetry of the cable structure can be eliminated. The unique physical position coordinate solution should satisfy the following:

(8) $$\left|x_{pd}-x_{B}|\approx v\cdot (t_{sB}-t_{0}\right)$$

where x_pd_ is the calculated axial coordinate of the PD source, x_A_ and x_B_ are the installation coordinates of Sagnac-A and Sagnac-B, respectively, and d_A_ and d_B_ are the calculated acoustic propagation distances from the PD source to the two Sagnac sensors. The system first extracts the acoustic time difference between the dual sensors, Δt = t_sB_ − t_sA_. For a single acoustic sensor, the calculated distance may correspond to two candidate positions on both sides of the sensor. The second Sagnac sensor is therefore used to verify the candidate position by checking the consistency between the calculated distances, the measured arrival-time difference, and the known spacing between Sagnac-A and Sagnac-B. If Δt ≈ (x_B_ − x_A_)/v, it proves that the acoustic wave passes through A and B sequentially from left to right, meaning the PD source must be to the left of A; in this case, x_pd_ = x_A_ − d_A_ is applied. If Δt is small, the PD source is located between the two sensors. In summary, the joint localization model proposed in this paper does not require solving complex nonlinear equation systems. It utilizes the HFCT to provide a unified electrical reference starting time; then utilizes Sagnac-A to acquire the absolute acoustic propagation delay T_A_ to accurately calculate the physical distance from the PD source to the sensor; and finally, incorporates the time-difference information from Sagnac-B to reduce directional ambiguity, thereby obtaining a unique axial localization result in the tested case.

### 2.4. Design of the Mandrel-Type Optical Fiber Sensor

The mandrel-type optical fiber sensor is the core sensitive structure in the Sagnac acoustic sensing unit [23]; its basic form involves tightly winding the sensing optical fiber around the surface of a cylindrical mandrel to form an optical fiber loop with a certain winding length. Let the radius of the mandrel be R, the height be H, and the total length of the sensing fiber be L. When the ultrasonic pressure P generated by a partial discharge acts on the bottom of the mandrel, the mandrel undergoes a minute elastic deformation, driving a change in the length of the optical fiber wound upon it, which ultimately causes optical phase modulation [24]. Existing research indicates that the sensitivity of a mandrel-type optical fiber probe directly depends on its material parameters and geometric dimensions.

For the mandrel-type optical fiber loop, the phase perturbation caused by the change in fiber length under ultrasonic excitation can be written as follows:

(9) Δϕ=βΔL

where β is the propagation constant, n is the refractive index of the fiber, and λ is the operating wavelength. If the mandrel is treated as a compressed elastic body, the increment in the length of the optical fiber loop can be further expressed as follows:

(10) $$\Delta L=\frac{2\pi N\mu HR^{2}}{REH-\mu E_{f}S_{f}N}P$$

where µ and E are the Poisson’s ratio and elastic modulus of the mandrel material, respectively; E_f_ and S_f_ are the elastic modulus and effective cross-sectional area of the optical fiber, respectively; and N is the number of winding turns. Since the number of winding turns approximately satisfies the following:

(11) $$N\approx \frac{L}{2\pi R}$$

Thus, the phase response model of the mandrel-type optical fiber loop can be obtained as follows:

(12) $$\Delta \phi =\frac{4\pi^{2}n\mu HR^{2}L}{\left(2\pi R^{2}EH-\mu E_{f}S_{f}L\right)\lambda }P$$

**Figure 4:**
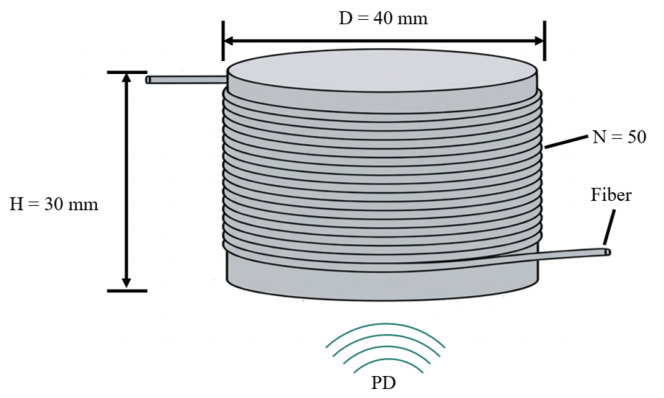
Schematic diagram of the mandrel sensor.

From the phase response model of the mandrel-type optical fiber loop above, it can be seen that the sensitivity of the loop is closely related to the mandrel material, the mandrel’s height, its radius, and the winding length. From a design perspective, the height of the mandrel should be appropriately reduced to enhance structural deformation under the same acoustic pressure; the winding length should be suitably increased to improve the phase accumulation effect, but excessive length can lead to increased transmission loss, a larger volume, and decreased winding feasibility. In contrast, the radius of the mandrel has a relatively minor impact on sensitivity, making it more suitable to be determined comprehensively based on installation space and packaging requirements. The formula indicates that sensitivity improves as the winding length increases and decreases as the mandrel height increases. To improve the ultrasonic detection sensitivity for partial discharge, an insulating material with a large Poisson’s ratio, a small elastic modulus, and low density should be selected for the mandrel, see Figure 4.

In the electro-acoustic joint detection system, during the process of the ultrasonic wave transmitting from the cable termination insulation layer into the sensor, the degree of interfacial acoustic impedance matching directly determines the transmittance of acoustic energy. According to the acoustic interface propagation theory, the acoustic energy transmission coefficient T depends on the acoustic impedances Z (the product of density and sound speed) of the two media [25]:

(13) $$T=\frac{4Z_{1}Z_{2}}{{\left(Z_{1}+Z_{2}\right)}^{2}}$$

where T is the acoustic energy transmission coefficient at the interface, and Z_1_ and Z_2_ are the acoustic impedances of the cable insulation and the mandrel material, respectively. The acoustic impedance is defined as the product of material density and sound velocity. The acoustic impedance of the cable’s XLPE insulation layer is known to be 1.7 × 10^6^ kg/(m^2^·s). If conventional plastic or air coupling is used, the impedance mismatch at the interface leads to severe acoustic reflection. The impedance of the photosensitive resin mandrel selected in this paper is 2.5 × 10^6^ kg/(m^2^·s); through calculation, its interfacial energy transmission coefficient reaches as high as 96.4%. In addition, by tightly winding the optical fiber onto the photosensitive resin mandrel, the mandrel undergoes minute radial deformation under the action of the acoustic pressure generated by the partial discharge, which translates into axial strain on the optical fiber. This structure utilizes the geometric gain effect of the mandrel, combined with the high phase sensitivity of the Sagnac interferometer to phase changes, significantly improving the system’s detection limit for weak PD acoustic emission signals.

The physical entity and structural parameters of the mandrel-type optical fiber loop sensor developed in this paper are shown in Table 1. In this study, a photosensitive resin with a Young’s modulus of 3 GPa and a Poisson’s ratio of 0.35 was selected as the base material for the mandrel structure, which was processed and fabricated using 3D printing technology. Benefiting from the micron-level manufacturing tolerances of high-precision photosensitive resin 3D printing, the sensing probes prepared in this paper maintain good physical consistency in their geometric dimensions, laying a reliable hardware foundation for subsequent array monitoring. A single-mode, small-diameter, bend-insensitive optical fiber was selected to reduce macroscopic bending losses during the winding process.

The primary response frequency near 33 kHz was selected as the designed resonant frequency of the mandrel-type Sagnac acoustic sensor rather than as a fixed characteristic frequency of all PD sources. For cable terminations, PD-induced acoustic signals can be effectively detected in the 20–40 kHz range [26], while low-frequency mechanical vibration is mainly distributed in the lower-frequency band. Therefore, a resonant response near 33 kHz is beneficial for enhancing weak PD acoustic signals and suppressing low-frequency vibration interference. Structurally, the resonant frequency of the mandrel-type sensor is governed by its equivalent stiffness and equivalent mass, which are mainly determined by the mandrel material, diameter, height, and fiber winding length. Based on the selected photosensitive resin material and the structural parameters listed in Table 1, the sensor was designed to have a dominant resonant response near 33 kHz.

## 3. Joint Detection Experiment

### 3.1. Amplitude–Frequency Characteristics Testing of the Optical Fiber Sensor

**Figure 5:**
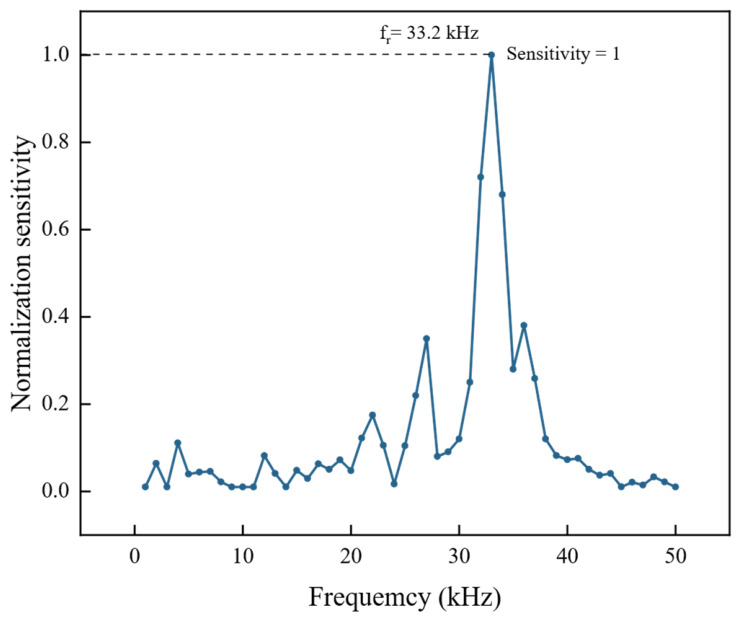
Amplitude–frequency test results.

To verify the response capability of the designed mandrel-type optical fiber loop sensor to partial discharge ultrasonic signals, and to provide an acoustic sensing characteristic basis for subsequent electro-acoustic synergistic partial discharge localization, this paper first constructed an amplitude–frequency characteristic testing platform for the sensor; the test results are shown in Figure 5. The testing system employs an arbitrary waveform generator to output a continuous sinusoidal sweep signal, which drives a standard piezoelectric ceramic transducer (PZT) via a power amplifier to serve as the ultrasonic emission source. The designed photosensitive resin mandrel-type optical fiber sensor and the piezoelectric ultrasonic transducer were tightly attached and fixed on a polyethylene testing platform; to ensure efficient acoustic wave coupling, a dedicated acoustic couplant was applied to the contact surface. In the experiment, the waveform generator was controlled to perform a step sweep in the range of 0–50 kHz, with a sweep step size set to 1 kHz, and the output voltage amplitude of the optical fiber sensing system at different driving frequencies was recorded. The test results are shown in Figure 5.

The amplitude–frequency result in Figure 5 is used to identify the dominant resonant response frequency of the mandrel-type Sagnac sensor under the present laboratory configuration. Since the purpose of this work is to verify the electro-acoustic localization method, the response is normalized to highlight the frequency-selective characteristic rather than reported as an absolute acoustic-pressure sensitivity. It should be noted that the narrow-band resonance may introduce waveform ringing and change the apparent waveform shape. Therefore, in the subsequent localization calculation, the acoustic arrival time is determined from the first distinguishable rising edge instead of the peak of the oscillatory waveform. A complete acoustic-pressure calibration, probe-to-probe repeatability test, temperature/coupling dependence analysis, and long-term stability evaluation will be further carried out in future engineering studies.

The test results indicate that the sensor exhibits its maximum normalized response near 33 kHz within the tested frequency range. This result confirms the dominant resonant response of the mandrel-type Sagnac sensor under the present laboratory configuration. It should be noted that this amplitude–frequency test is intended as a preliminary characterization of the developed sensor for the present electro-acoustic localization experiment. The response is normalized to highlight the frequency-selective characteristic, rather than calibrated in acoustic-pressure units. Therefore, the present result does not constitute a complete metrological characterization of the sensor. Further evaluation of acoustic-pressure sensitivity, bandwidth and quality factor, probe-to-probe repeatability, temperature dependence, coupling dependence, and long-term stability will be required in future work. Temperature and external vibration may affect the sensor response mainly by changing the mechanical properties of the mandrel, the acoustic coupling state, and the background noise level. In the present laboratory test, the narrow-band resonant response near 33.2 kHz helps suppress low-frequency mechanical vibration components, and all photoelectric conversion modules were shielded to reduce external interference. However, the current study focuses on the feasibility of electro-acoustic joint localization under controlled conditions. The quantitative influence of temperature variation and long-term external vibration will be further evaluated in future field tests.

### 3.2. Electro-Acoustic Synergistic PD Detection and Localization

**Figure 6:**
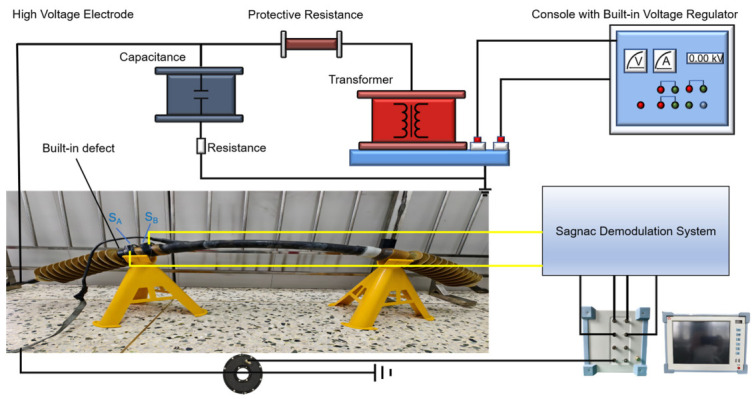
Electro-acoustic synergistic partial discharge detection and localization experimental platform.

To systematically evaluate the joint localization efficacy of the HFCT and the dual Sagnac optical fiber sensing array, this study constructed an electro-acoustic synergistic partial discharge testing platform on a cable termination model (as shown in Figure 6). Internally, a built-in defect model within the termination stress cone was used to simulate an air-gap discharge defect inside the termination. In this study, the air-gap defect was used as a representative and controllable PD source to verify the electro-acoustic localization method. The purpose of this work is to evaluate the localization performance of the proposed system, rather than to classify different PD defect types according to their acoustic spectra. Regarding the spatial coordinate system setup, the starting end of the cable stress cone was set as the one-dimensional reference origin (x = 0 cm). The HFCT was coupled to the system’s grounding return line, and two sets of Sagnac optical fiber sensing units (Sagnac-A and Sagnac-B) with highly consistent characteristic parameters were attached to fixed points on the termination surface. A high-speed digital oscilloscope (sampling rate 2 GS/s) was utilized for synchronous triggered acquisition. To ensure signal integrity, all photoelectric conversion modules were placed inside an electromagnetic shielding box to maximally suppress laboratory background noise.

**Figure 7:**
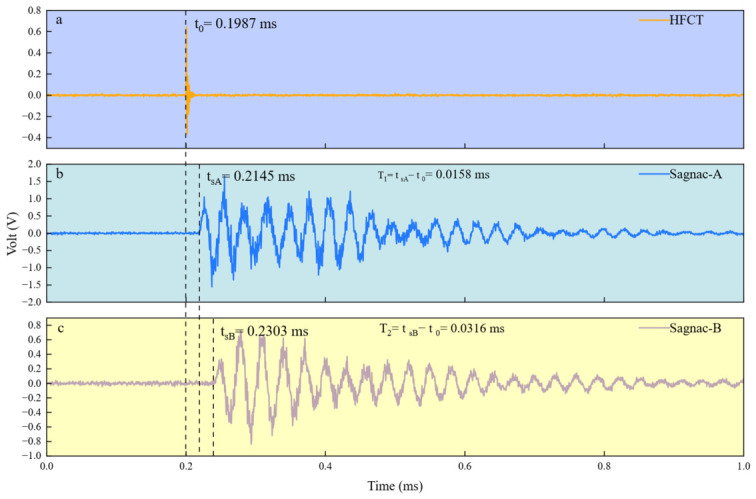
Synchronous localization waveform diagrams: (**a**) HFCT current pulse signal; (**b**) acoustic response of Sagnac-A; (**c**) acoustic response of Sagnac-B.

The synchronous time-domain response curves of the multi-source sensors to a single partial discharge event are shown in Figure 7. By applying a 12 kV power-frequency voltage to excite a stable partial discharge, at the transient moment the discharge occurs, the HFCT captures the partial discharge current pulse first, relying on its wide-band electromagnetic response (Figure 7a). Because the propagation speed of electromagnetic waves is far higher than that of ultrasonic waves, the electrical signal transmission delay can be ignored in microsecond-level calculations; the rising edge of this pulse signal is precisely extracted and serves as the absolute time zero point for the occurrence of the PD event (t_0_ = 0.1987 ms). At the acoustic response level, constrained by the transit time of acoustic waves in multi-layer media such as cross-linked polyethylene (XLPE), the two sets of optical fiber sensing systems exhibit a significant time delay. Benefiting from the strong resonant amplification effect designed at 33 kHz in the sensor’s mandrel structure, the targeted high-frequency acoustic energy is effectively selected and amplified, enabling the system to obtain a relatively clear acoustic response under the laboratory electromagnetic background. As shown in Figure 7b,c, the initial rising edges of the ultrasonic waveforms can be identified from the background noise, which is beneficial for arrival-time extraction. Sagnac-A and Sagnac-B captured the rising edges of the pulse signals at t_sA_ = 0.2145 ms and t_sB_ = 0.2303 ms, respectively, The main uncertainty sources in the electro-acoustic localization process are summarized in Table 2.

Before the localization calculation, the timing consistency of the electro-acoustic acquisition system was checked under the same acquisition configuration, and the fixed channel delays were compensated in the time-of-flight calculation. The fixed delays introduced by the HFCT channel, photodetectors, Sagnac demodulation links, coaxial cables, and oscilloscope channels were treated as channel-dependent constants. Therefore, the corrected time values can be expressed as t_HFCT_ = t_HFCT,m_ − τ_HFCT_, t_SA_ = t_SA,m_ − τ_SA_ and t_SB_ = t_SB,m_ − τ_SB_, where t_m_ is the measured time and τ is the fixed delay of the corresponding channel. The acoustic time-of-flight values were then obtained as T_A_ = t_SA_ − t_HFCT_ and T_B_ = t_SB_ − t_HFCT_. The acoustic velocity of 1900 m/s was used as the effective axial propagation velocity in the tested cable-termination structure. The main uncertainty sources include acoustic velocity deviation, sensor positioning error, oscilloscope timing resolution, channel skew, acoustic onset extraction, and coupling-state variation. It should be noted that this discussion is intended as an uncertainty-source analysis rather than a complete quantitative uncertainty budget. A complete metrological uncertainty budget would require repeated measurements and numerical evaluation of each uncertainty component, which will be carried out in future work.

The oscilloscope sampling rate was 2 GS/s, corresponding to a sampling interval of 0.5 ns, which is much smaller than the microsecond-level acoustic propagation delay. For the effective acoustic velocity of 1900 m/s, a timing deviation of 1 μs corresponds to a distance deviation of approximately 1.9 mm. Therefore, the dominant error sources are mainly related to effective acoustic velocity, sensor positioning, acoustic onset extraction, and coupling-state variation rather than oscilloscope timing resolution. The electromagnetic shielding box and the 33.2 kHz narrow-band response were used to reduce electromagnetic interference and low-frequency vibration disturbance.

The oscillatory components in the Sagnac-A and Sagnac-B waveforms are associated with the narrow-band resonant response of the mandrel-type sensor, verified by the amplitude–frequency sweep test in Figure 5; in Figure 7, these waveforms are mainly used for acoustic arrival-time extraction rather than for independent frequency-response characterization.

Based on the extracted time points, the acoustic time-of-flight values can be estimated as T_A_ = t_sA_ − t_0_ = 0.2145 ms − 0.1987 ms = 15.8 μs and T_B_ = t_sB_ − t_0_ = 0.2303 ms − 0.1987 ms = 31.6 μs. With the effective axial acoustic velocity of 1900 m/s, the corresponding propagation distances are d_A_ = v_TA_ = 30.02 mm and d_B_ = v_TB_ = 60.04 mm, respectively. These calculated values agree with the distance results obtained from the experimental localization analysis, namely 3.002 cm for Sagnac-A and 6.004 cm for Sagnac-B. The comparison indicates that the experimentally extracted electro-acoustic time delays are consistent with the theoretical time-of-flight estimation under the tested configuration. The remaining position deviation is mainly attributed to the effective acoustic velocity, sensor positioning, acoustic onset extraction, and coupling-state variation.

**Figure 8:**
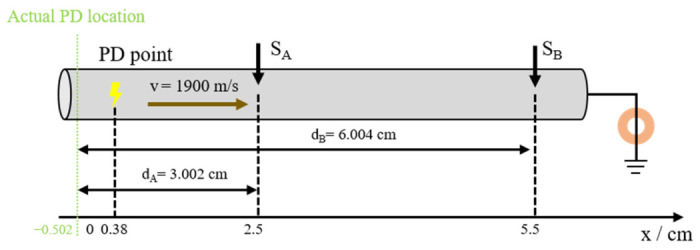
Schematic diagram of the actual distance from the discharge point to the sensors.

Based on the extracted electro-acoustic time features, the calculation logic and physical mapping of the spatial localization are shown in Figure 8. In the experiment designed in this paper, the physical installation coordinate of Sagnac-A is x_A_ = 2.5 cm, and the physical installation coordinate of Sagnac-B is x_B_ = 5.5 cm. The measured electro-acoustic time delay difference is Δt_A_ = t_sA_ − t_0_. Combined with the calibrated sound velocity v in the cable medium, the absolute physical distance d_A_ from the PD source to Sensor A can be calculated as 3.002 cm. Because the redundant configuration of the dual sensing array (Sagnac-A and Sagnac-B) eliminates the linear directional ambiguity of one-dimensional acoustic wave propagation, as shown in Figure 8, the unique spatial coordinate of the partial discharge deduced inversely from Sensor A is x_pd_ = x_A_ − d_A_ = −0.502 cm. Similarly, the spatial coordinate verified and calculated by Sensor B is x_calc_ = x_B_ − d_B_= −0.506 cm, showing a highly consistent dual-sensor verification. A comparison between the experimental results and the physical layout indicates that the absolute error between this calculated coordinate and the preset true defect coordinate inside the termination is 5 mm. In the tested case, the calculated distances from the PD source to Sagnac-A and Sagnac-B were consistent with the known sensor spacing, indicating that the PD source was located outside the sensor pair on the left side of Sagnac-A and that the pseudo-solution on the opposite side could be excluded. Figure 8 presents the spatial relationship between the calculated PD position and the sensor layout. This result verifies the feasibility of using the HFCT electrical reference together with dual-Sagnac acoustic arrival times for axial PD localization in the tested cable-termination structure.

In the tested case, the calculated distances from the PD source to Sagnac-A and Sagnac-B were consistent with the known sensor spacing, indicating that the representative PD source was located outside the sensor pair on the left side of Sagnac-A. However, this experiment only validates the discrimination logic under the present sensor arrangement and defect position. Additional tests with PD sources located between the sensors and outside the sensor pair on both sides are still required to fully evaluate the general applicability of the direction-discrimination rule.

The repeatability of the electro-acoustic localization result is mainly affected by the stability of the HFCT trigger point, the clarity of the first acoustic arrival, sensor positioning, acoustic coupling, effective acoustic velocity, and background noise. In the present laboratory configuration, the HFCT pulse and the Sagnac acoustic rising edges can be clearly distinguished from the recorded waveforms, which provides the basis for stable time-feature extraction in the tested case. However, since this work mainly focuses on verifying the feasibility of the HFCT-assisted dual-Sagnac localization principle, a complete statistical evaluation involving a larger number of repeated PD events, different defect positions, sensor spacings, discharge intensities, and field noise conditions will be carried out in future work.

## 4. Conclusions

Aiming at the engineering requirements for high-sensitivity detection and localization feasibility of partial discharge in high-voltage cables, this paper proposes and verifies an electro-acoustic synergistic localization method integrating a high-frequency current transformer (HFCT) and a dual-Sagnac optical fiber interferometer. The main conclusions are as follows:

(1) The designed photosensitive resin mandrel structure achieves acoustic impedance matching between the sensor and the cable’s XLPE insulation layer, significantly increasing the theoretical energy transmittance and providing physical gain for the capture of weak PD signals. Through structural parameter design and amplitude–frequency test verification, the sensor is able to generate a significant acoustic resonant amplification effect at 33 kHz. The present sensor characterization should also be regarded as a preliminary normalized amplitude–frequency evaluation, and complete metrological calibration involving acoustic-pressure sensitivity, bandwidth, repeatability, temperature/coupling dependence, and long-term stability will be carried out in future work.
(2) A joint localization logic is proposed, employing an electrical signal to establish the time benchmark and dual acoustic signals for ranging and direction finding. Utilizing the rising edge of the pulse signal captured by the HFCT as a reference effectively overcomes the bottleneck of a single fiber-optic acoustic system lacking a time reference. Meanwhile, utilizing the acoustic time-difference information from the dual Sagnac probes helps reduce the ambiguity of the one-dimensional acoustic wave propagation direction under the tested configuration in the cable, achieving a more complete localization logic. This method not only solves the problem of a single acoustic sensor being unable to perform localization but also improves the reliability of the localization calculation under the tested configuration compared to dual-acoustic sensor localization, due to the introduction of the absolute time zero point calibrated by the electrical signal.

Localization verification was conducted based on a cable termination physical model. Experimental results indicate that, benefiting from the steep signal starting edge brought by the 33 kHz narrow-band resonance, the system can accurately identify the acoustic-electrical time difference. For the representative built-in air-gap defect under the tested laboratory cable-termination configuration, the absolute error between the calculated PD position and the preset physical position was 5 mm. This result demonstrates the feasibility of the proposed HFCT-assisted dual-Sagnac electro-acoustic localization concept. However, this value should not be regarded as a generally validated system accuracy, because broader validation involving multiple defect positions, sensor spacings, discharge intensities, repeated PD events, and field noise conditions is still required.

## Figures and Tables

**Table 1:** Key structural parameters of the mandrel-type optical fiber loop sensor.

Parameter Name	Symbol	Value	Unit
Mandrel Outer Diameter	D	40	mm
Mandrel Height	H	30	mm
Number of Winding Turns	N	50	turns
Total Winding Length	L	6.28	m
Designed Main Resonant Frequency of Mandrel	f_r_	33.2	kHz

**Table 2:** Main uncertainty sources in electro-acoustic localization.

Source	Influence on Localization
Effective acoustic velocity	Directly affects the conversion from time-of-flight to distance
Sensor positioning	Introduces coordinate error of Sagnac-A and Sagnac-B
Oscilloscope timing resolution	Affects the precision of arrival-time reading
Channel skew and fixed delay	Causes systematic time offset if not compensated
Onset-time extraction	Influences the determination of HFCT and acoustic rising edges
Acoustic coupling state	Affects acoustic amplitude and the clarity of the first arrival

## Data Availability

The original contributions presented in this study are included in the article. Further inquiries can be directed to the corresponding author.
